# Surgical Intervention for Isolated Tricuspid Valve Endocarditis—Refining Patients' Selection

**DOI:** 10.1055/s-0045-1808059

**Published:** 2025-06-02

**Authors:** Ali Hage, Rami Abazid, Fadi Hage, Shevan Bladia, Linrui Guo, Nikolaos Tzemos

**Affiliations:** 1 Division of Cardiac Surgery, Department of Surgery, London Health Sciences Centre, Western University, London, Ontario, Canada; 2 Department of Medicine, Northern Ontario Medical School University, Sault Ste Marie, Ontario, Canada; 3 College of Health Sciences, Western University, London, Ontario, Canada; 4 Division of Cardiology, Department of Medicine, London Health Sciences Centre, Western University, London, Ontario, Canada

**Keywords:** tricuspid valve, infective endocarditis, tricuspid regurgitation

## Abstract

**Background:**

In this study, we analyzed various clinical and imaging factors of patients with isolated tricuspid valve infective endocarditis (TVIE) who have undergone surgical intervention, and assessed short- and long-term outcomes after surgery.

**Methods:**

We retrospectively enrolled 26 patients diagnosed with definite isolated TVIE and underwent surgical intervention between February 2004 and August 2019. We collected patients' demographics, preoperative and postoperative data. The primary outcomes were death and a composite of the following: death, readmission with right-sided heart failure, or recurrent endocarditis.

**Results:**

A total of 29 isolated tricuspid valve surgical interventions were performed on 26 patients. The mean age was 38.6 ± 12.3 years. In total, 22/29 (75.8%) of TVIE were related to *Staphylococcus aureus* and 4/29 (13.8%) were secondary to fungal infection. During a follow-up of 5.4 ± 3.7 years, there were 9 (34.6%) deaths and 15 (57.7%) composite outcomes. Multivariable Cox regression analysis showed that male sex (hazard ratio [HR]: 16.68, 95% confidence interval [CI]: 1.63–170.34, *p* = 0.018) and intravenous drug users (IVDU) (HR: 25.66, 95% CI: 1.87–352.79, *p* = 0.015) are significantly associated with increase death; on the other hand, higher level of preoperative hemoglobin and preoperative left ventricular ejection fraction (LVEF) was found to have decreased hazard of death: HR: 0.90, 95% CI: 0.82–0.99, *p* = 0.033 and HR: 0.92, 95% CI: 0.86–0.98, *p* = 0.013, respectively.

**Conclusion:**

In our institution, surgical intervention for isolated TVIE has a mortality rate of 34.6%. Men, a history of IVDU, lower preoperative hemoglobin levels, and reduced LVEF were significant predictors of postsurgical mortality. Earlier surgical intervention for TVIE before the development of anemia or impaired LV systolic function may have a potential survival benefit.

## Introduction

Isolated tricuspid valve infective endocarditis (TVIE) is increasing in incidence due to the rising number of intravenous drug users (IVDU), cardiac device implantation, and long-term use of central venous access catheters. 1 Although TVIE accounts for only 2.5% of all cases of primary and secondary forms of isolated severe tricuspid regurgitation (TR), 2 other studies have reported higher prevalence rates. For instance, one study found a 22% prevalence of infective endocarditis (IE) among patients undergoing surgery in the United States, with higher rates observed in valve replacement procedures (42%) compared with valve repair (12%). 3 Unlike left-sided IE, right-sided IE is less invasive and rarely results in cavities or abscess formation, 4 and thus the role of surgery for right-sided IE is not very well defined nor adequately explored. 4 In fact, for right-sided IE, the general indications for surgery are failure to control the infection despite appropriate medical therapy, resulting in septic pulmonary embolism, increased pulmonary vascular resistance, and consequently, inability to tolerate TR and the resultant congestive right heart failure. 5 Few studies reported better survival postsurgical intervention in patients with primary TR than those with secondary TR 2 6 ; however, a very recent report showed that late mortality (30 days after tricuspid valve [TV] surgery) was significantly higher with TVIE compared with other etiologies. 7 Moreover, the early postoperative survival did not differ between IVDU and non-IVDU TVIE who underwent valve surgery. 8

While the timing of surgery remains uncertain, it is ideal to sterilize the valve and surrounding tissues surgically. However, delaying surgery excessively may increase the risk of complications. When opting for surgical management, current guidelines favor valve repair over replacement, especially in cases where there is no notable leaflet destruction. 5 7 9 10 Additionally, percutaneous vegetation debulking with the aspiration thrombectomy device (AngioVac system) can serve as an alternative treatment option for patients at high surgical risk or those presenting with severe septic shock. 11 In this study, we aim to assess various clinical and imaging factors among individuals who have undergone cardiac surgical intervention for isolated TVIE and to investigate their short- and long-term outcomes.

## Methods

### Study Design

All patients diagnosed with isolated TVIE who underwent surgical intervention between February 2004 and August 2019 at London Health Sciences Centre, Ontario, Canada, were enrolled in a registry and subsequently followed up. The study included all patients aged 18 years or older, presenting with a definite diagnosis of IE solely affecting the TV. These patients had not responded to medical therapy for IE and required surgical intervention on the TV.

The collected data included patients' demographics, preoperative comorbidities, clinical, microbiological, and echocardiographic findings at the time of presentation with TVIE, medical therapy for TVIE, indications for TV intervention, operative procedure, postoperative course in hospital, long-term complications, readmissions, need for reintervention, and survival. Clinical outcomes were collected and censored until August 2019.

All patients underwent clinical follow-up within 6 weeks after surgery, with additional follow-up visits scheduled at 3 to 6 months postoperatively, and subsequently, on an annual basis. Furthermore, all patients underwent postoperative echocardiographic assessments within 3 months following surgery, followed by subsequent annual assessments.

During the study period (February 2004–August 2019), primary outcomes of interest were cardiac death and a composite of the following: cardiac death, readmission with right-sided heart failure or recurrent endocarditis, and need for reintervention.

### Statistical Analysis

Data were analyzed using Stata version 16.0 (StataCorp LLC, College Station, Texas, United States). *We used Shapiro–Wilk's test to assess the distribution of the data.* Continuous variables were compared using unpaired *t* -tests (when data were normally distributed) or Mann–Whitney's *U* test (when data were skewed). Categorical variables were analyzed with either the Pearson's chi-square or the Fisher's exact test. A probability value ( *p* ) of <0.05 was considered statistically significant. To elucidate the association of various preoperative clinical and echocardiographic predictors on event's occurrence, we performed multivariable Cox regression. Because of the small size of the cohort, we built two separate regression models for each of the outcomes of interest: one regression model included five preoperative clinical covariates (sex, IVDU, cerebrovascular disease, preoperative creatinine, and preoperative hemoglobin) and another regression model included four preoperative echocardiographic covariates (left ventricular ejection fraction [LVEF] [%], right ventricular [RV] size, RV function, and TR degree). These covariates were clinically judged to be of prognostic significance. The results of the multivariable Cox regression models were displayed graphically using Cox proportional hazards regression curves. We verified the proportional hazard assumption with Schoenfeld residuals and using visual inspection of the log curves.

## Results

### Preoperative Baseline Characteristics

A total of 29 isolated TV surgical interventions were performed on 26 patients, constituting the study population. The mean age at the time of the first surgical intervention was 38.6 ± 12.3 years. Among the patients, 19 (73.1%) had a history of IVDU. All 26 patients had isolated native TVIE, with three patients requiring reintervention for their TV. Other baseline characteristics and comorbidities prior to surgical intervention are outlined in Table 1 . The primary indication for isolated TV surgical intervention was either uncontrolled sepsis despite microorganism-specific antibiotic therapy in 23 (79.3%) cases or significant and symptomatic heart failure in the remaining 6 (20.7%) cases. Furthermore, one case (3.4%) presented with pulmonary embolism, while another (3.4%) experienced paradoxical septic embolism in the presence of a patent foramen ovale. Preoperative echocardiographic assessments revealed that at least moderate TR was present in 24 (82.7%) prior to surgical intervention, and other echocardiographic findings are detailed in Table 1 . The mean LVEF at 1-year postoperative follow-up for surviving patients was 51 ± 8%.

**Table 1:** Patients' baseline characteristics

Preoperative clinical and echocardiographic characteristics	
Age at the time of the first surgical intervention, y (mean ± SD)	39 ± 12
Male, *n* (%)	16 (53.9)
*Staphylococcus aureus* infection, *n* (%)	22 (75.8)
Fungal infection, *n* (%)	4 (13.8)
Infected pacemaker leads, *n* (%)	2 (6.9)
Intravenous drug use, *n* (%)	19 (73.1)
Right-sided heart failure, *n* (%)	20 (68.9)
Anemia, *n* (%)	25 (86.2)
Preoperative creatinine, µmol/L (mean ± SD)	127 ± 147
Congestive heart failure, *n* (%)	17 (65.4)
Preoperative dialysis, *n* (%)	6 (23.1)
Use of diuresis, right heart catheterization, *n* (%)	26 (100)
Nephropathy, *n* (%)	13 (50)
Congestive hepatopathy, *n* (%)	17 (65.4)
Lower limb edema, *n* (%)	24 (92.3)
Median preoperative LOS (IQR), d	11 (3–18)
Median antibiotics duration (IQR), d	22 (14–42)
Right heart catheterization, *n* (%)	15 (57.7)
Vegetation size, mm (mean ± SD)	21 ± 11
Preoperative ≥ moderate tricuspid regurgitation, *n* (%)	20 (76.9)
Preoperative ≥ moderate right ventricular dilatation, *n* (%)	11 (42.3)
Preoperative ≥ moderate right ventricular dysfunction, *n* (%)	2 (7.7)
Preoperative mean left ventricular ejection fraction %, (mean ± SD)	53 ± 11
Pulmonary artery pulsatility index: systolic pulmonary artery pressure − diastolic pulmonary artery pressure/right atrial pressure	1.63 ± 0.6

Abbreviations: IQR, interquartile range; LOS, length of stay; SD, standard deviation.

### Surgical Intervention

One of three surgical operations was performed on these patients presenting with isolated TVIE: TV repair/vegetectomy in 19 (65.5%) cases, bioprosthetic TV replacement in 8 (27.6%) cases, or tricuspid valvectomy in 2 (6.9%) cases. During follow-up, three patients underwent reintervention due to recurrent endocarditis caused by *Staphylococcus aureus* . The first recurrence occurred after 7 months postoperation, the second recurrence after 13 months, and the third recurrence after 26 months. In two of these cases, the initial surgical intervention involved TV repair, followed by subsequent TV replacement. In the third case, the initial surgery was TV replacement with subsequent tricuspid valvectomy.

### Outcomes

The mean follow-up duration was 5.4 ± 3.7, with all patients having regular follow-up visits. The maximum follow-up duration was 12.2 years. During this period, nine patients (34.6%) died. Specifically, three deaths occurred within 30 days of surgery, while five occurred within 6 months of surgery. Additionally, Table 2 provides a summary of other in-hospital postoperative complications.

**Table 2:** Early postoperative outcomes

30-day postoperative outcomes	
Death, *n* (%)	3 (11.5)
Stroke, *n* (%)	1 (3.9)
Intra-aortic balloon pump, *n* (%)	1 (3.9)
Mediastinitis, *n* (%)	0 (0)
New dialysis, *n* (%)	7 (26.9)
Sepsis, *n* (%)	8 (30.8)
Respiratory failure, *n* (%)	3 (11.5)
Reoperation for bleeding, *n* (%)	2 (7.7)
Median intensive care unit LOS (IQR), d	2 (1–5)
Median hospital LOS (IQR), d	14 (7–19)

Abbreviations: IQR, interquartile range; LOS, length of stay.

### Time-to-Event Analysis

**Fig. 1:**
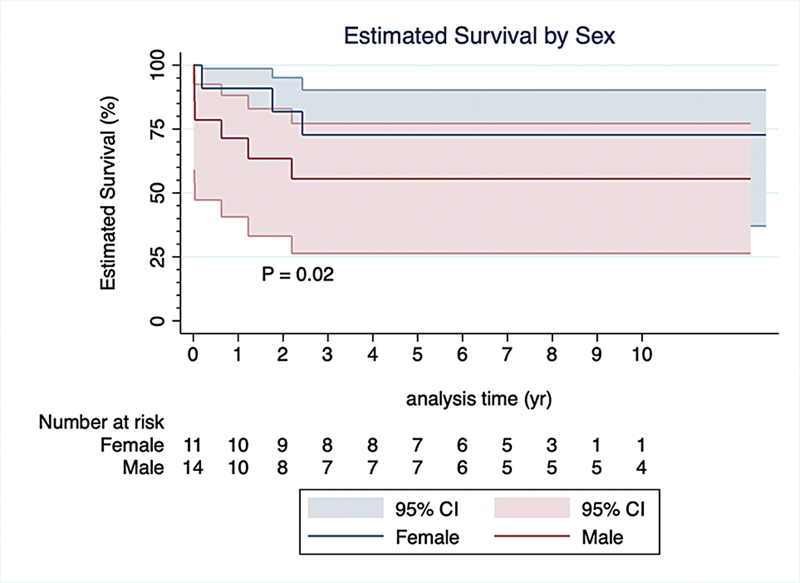
Cox proportional hazard curves comparing survival between females and males. CI, confidence interval.

**Fig. 2:**
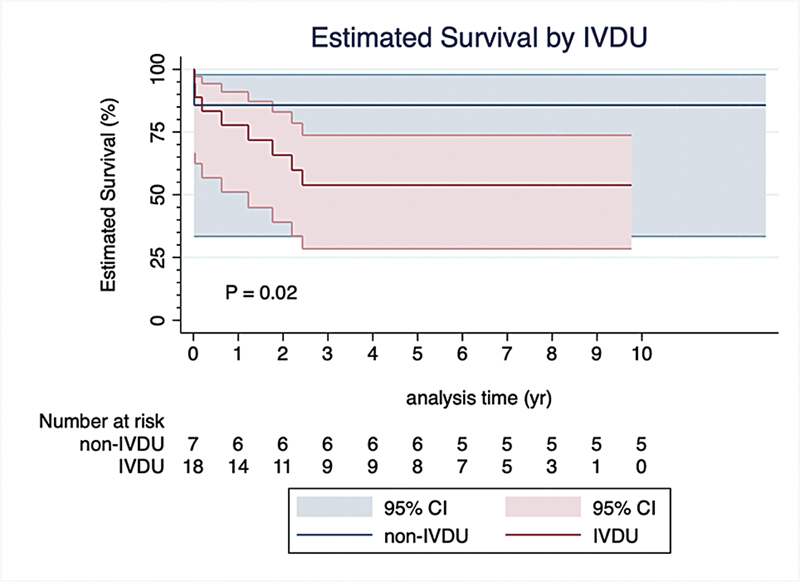
Cox proportional hazard curves comparing survival between IVDU and non-IVDU. CI, confidence interval; IVDU, intravenous drug users.

A time-to-event analysis was conducted to identify potential clinical and echocardiographic associated with death. Male sex and IVDU were the only two factors associated with increased hazard of death ( Table 3 ; Figs. 1 and 2 ). In contrast, patients with a preoperative hemoglobin level greater than 10.0 mg/dL and those with normal preoperative LVEF demonstrated a decreased hazard of death. Additionally, factors such as cerebrovascular disease, preoperative elevated creatinine, preoperative moderate or worse RV systolic dysfunction, and preoperative moderate or greater RV dilatation did not significantly influence the hazard of death ( Table 3 ).

**Table 3:** (A) Time-to-event analysis to identify potential associates of death and (B) time-to-event analysis to identify potential associates of the composite outcome of death, readmission due to right-sided heart failure, or recurrent endocarditis

	A			B		
Variable	Hazard ratio	95% CI	*p* -Value	Hazard ratio	95% CI	*p* -Value
Male sex	16.68	1.63–170	0.018	15.20	2.35–98.22	0.004
IVDU	25.66	1.87–352	0.015	37.82	3.77–379.60	0.002
Preoperative hemoglobin >10.0 mg/dL	0.90	0.82–0.99	0.033	0.89	0.83–0.96	0.002
Normal preoperative LVEF	0.92	0.86–0.98	0.013	0.90	0.84–0.96	0.002
Cerebrovascular disease	2.25	0.17–29.90	0.54	8.70	0.87–87.17	0.066
Preoperative elevated creatinine	0.99	0.98–1.00	0.052	1.00	0.99–1.00	0.063
Preoperative RV dysfunction	2.66	0.22–31.83	0.44	1.07	0.11–10.09	0.06
Preoperative RV dilation	1.13	0.14–8.84	0.90	0.32	0.08–1.28	0.11

Abbreviations: CI, confidence interval; IVDU, intravenous drug users; LV, left ventricular; LVEF, left ventricular ejection fraction; RV, right ventricular.

**Fig. 3:**
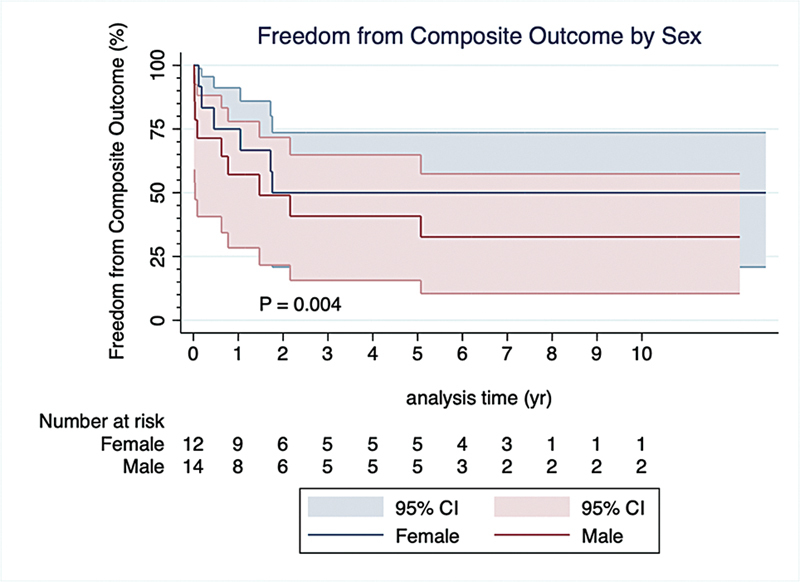
Cox proportional hazard curves comparing freedom from the composite outcome of death, readmission with right-sided heart failure or recurrent endocarditis, and need for reintervention, between females and males. CI, confidence interval.

**Fig. 4:**
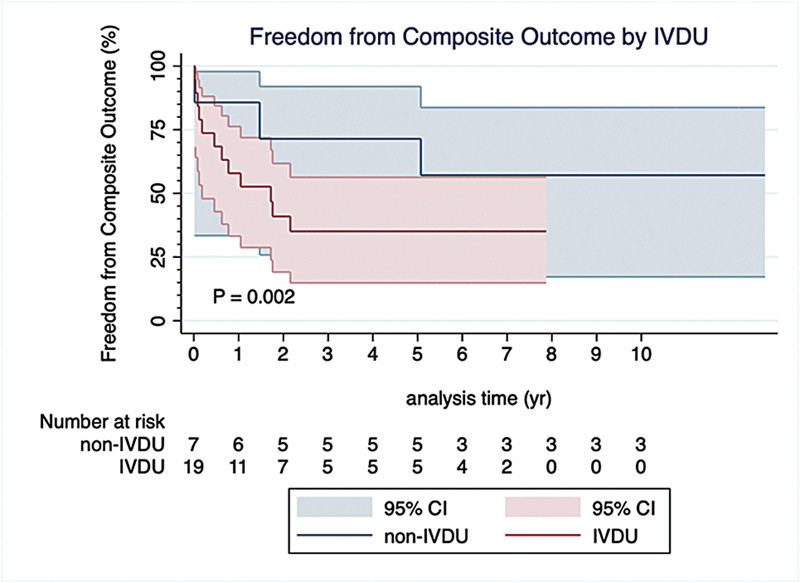
Cox proportional hazard curves comparing freedom from the composite outcome of death, readmission with right-sided heart failure or recurrent endocarditis, and need for reintervention, between IVDU and non-IVDU. CI, confidence interval; IVDU, intravenous drug users.

These results were consistent when examining the composite outcome of death, readmission with right-sided heart failure or recurrent endocarditis, and the need for reintervention. The composite outcome occurred in 15 (57.7%) patients. Male sex ( Fig. 3 ; Table 3 ) and IVDU ( Fig. 4 ; Table 3 ) were the only two factors associated with increased hazard of the composite outcome. Normal preoperative hemoglobin and normal preoperative LVEF were both associated with decreased hazard of the composite outcome. Cerebrovascular disease, preoperative elevated creatinine, preoperative ≥moderately impaired RV systolic function, and preoperative ≥ moderately dilated did not influence the hazard of the composite outcome ( Table 3 ).

## Discussion

In this study, we investigated the clinical and echocardiographic factors associated with postoperative mortality and morbidity of patients undergoing surgical intervention for isolated TVIE. Our findings suggest that male patients and those with a history of IVDU are at significantly higher risk of death and complications in the short- and intermediate-term following isolated TV surgical intervention. In contrast, patients presenting with preserved left ventricular function appear to be more protected from postoperative mortality and morbidity.

The incidence of TVIE is lower compared with left-sided IE and is estimated to account for 10% of all cases of IE. The majority of patients with TVIE are successfully treated with antibiotics, and only a small proportion require surgical intervention, either due to failed medical management or after secondary complications such as septic pulmonary emboli. 12 Much of the debate surrounding surgical intervention for TVIE revolves around whether to repair or replace the TV. In terms of survival, current evidence suggests a similarity between both techniques. For instance, in a propensity-score matched cohort study by Moraca et al, similar 10-year survival rates were reported between both surgical options, with a 49% survival for the replacement group and a 66% survival for the repair group ( *p* = 0.66). 13 Therefore, regardless of the surgical approach, survival outcomes tend to be poor, underscoring the importance of identifying predictors of long-term survival to enhance patient outcomes. Additionally, Alnajar et al reported no significant differences in outcomes between patients undergoing TV interventions (repair or replacement) for TR associated with IE and those with TR unrelated to IE. 3

We found that male patients are at an increased risk of mortality and morbidity. Historically, men formed the majority of those presenting with TVIE. One potential factor contributing to the sex disparity in mortality outcomes is the higher prevalence of IVDU among male patients of our study cohort, which was identified as another risk factor associated with increased hazard of mortality and morbidity. In this patient population, it is believed that the TV infection occurs on a valve that has already been damaged by previous episodes of infection. 6 Regrettably, most of these patients will resume drug injection soon after their surgeries. 14 Similar to the report of Wang et al, we found that hemoglobin levels of < 10 mg/dL were associated with higher hazards of complications. This is explained by the fact that anemia is a surrogate marker of a more advanced systemic disease.

Regarding echocardiographic markers, the presence of LV dysfunction appeared to influence long-term survival and complications. As for the RV, there was a strong trend between suggesting that RV dilatation and dysfunction were associated with worse outcomes. However, it is important to interpret these findings within the context that most patients with LV dysfunction had concomitant RV dilatation and dysfunction. It is well known that the majority of patients presenting with TVIE will undergo a full course of antibiotics treatment, which lead to one of two outcomes: (1) complete resolution of the infection without the need for surgery; or (2) sterilization of the infection, thus avoiding the use of prosthetic materials in an infected field during surgery. However, an important question arises regarding the optimal timing of surgery. There is a subset of patients who may benefit from earlier surgery, potentially before the onset of RV or LV failure. This raises the challenge of determining which patients will benefit from “delayed” surgery after completing a full course of antibiotics, and which patients require “early” surgery (i.e., before completing the antibiotics regimen) to prevent the progression to ventricular dysfunction. The current literature about the timing of surgery mainly focuses of left-sided lesions and generally support the notion that early surgery reduces the risk of death and systemic embolization. 15 However, in cases where the patients present with ischemic or hemorrhagic stroke, delaying surgery for 1 to 2 or >3 weeks, respectively, is warranted to reduce the risk of mortality and postoperative neurologic complications. 16

In our study, only three patients (11.5%) required reintervention, which was slightly higher than the reoperation rate reported by Alnajar et al using minimally invasive isolated TV interventions. 3 However, in Alnajar et al's cohort, only 7.8% of cases were due to IE, which likely explains their slightly lower reoperation rate. We were unable to identify specific factors associated with this complication. However, what was evident is that during the second intervention, there was an “escalation” of the approach: patients who initially underwent repair later required a valve replacement, and the patient who initially had a replacement subsequently needed a valvectomy. Current evidence suggests that TV repair is superior to replacement in terms of reducing the risk of reintervention. A systematic review and meta-analysis by Yanagawa et al found that, despite similar long-term survival between TV repair and replacement, there was a higher freedom from recurrent TVIE and reintervention when the TV was initially repaired. 17 In our study, we found that anemia (hemoglobin <10 g/dL) and reduced LVEF were associated with increased postsurgical mortality, and thus, we suggest considering these factors as markers for earlier surgical intervention in patients with TVIE.

Our study reflects the reality of Canadian urban areas heavily affected by the high rate of intravenous drug abuse, where patients often present late to the hospital with uncontrolled infection or fail to maintain a drug-free lifestyle after surgery. This results in a recurrence of the same issues, highlighting the challenges of managing this patient population.

## Study Limitations

Our study has some limitations. Given the small sample size, single-center design, and the high prevalence of IVDU in our study cohort, we believe that our results may not be directly applicable to other patient populations, particularly those with severe TR and IE in different subgroups of patients. Our cohort had a short duration of follow-up, and a longer follow-up is required to better investigate the long-term outcomes.

## Conclusion

Among patients with isolated TVIE, men and those with a history of IVDU are found to have a significantly higher risk of death, short- and intermediate-term complications following TV surgical intervention. In contrast, patients presenting with preserved left ventricular function and those with higher preoperative hemoglobin levels seem to be protected from postoperative mortality and morbidity.

## References

[1] Gaca J G Sheng S Daneshmand M Current outcomes for tricuspid valve infective endocarditis surgery in North America Ann Thorac Surg 2013 96 04 1374 1381 23968767 10.1016/j.athoracsur.2013.05.046

[2] Wang T KM Akyuz K Mentias A Contemporary etiologies, outcomes, and novel risk score for isolated tricuspid regurgitation JACC Cardiovasc Imaging 2022 15 05 731 744 34922866 10.1016/j.jcmg.2021.10.015

[3] Alnajar A Arora Y Benck K N Isolated tricuspid valve repair versus replacement: predictors of mortality on the national level Innovations (Phila) 2023 18 01 58 66 36802966 10.1177/15569845231153127

[4] Hussain S T Shrestha N K Witten J Rarity of invasiveness in right-sided infective endocarditis J Thorac Cardiovasc Surg 2018 155 01 54 610 28951083 10.1016/j.jtcvs.2017.07.068

[5] Pettersson G B Hussain S T Current AATS guidelines on surgical treatment of infective endocarditis Ann Cardiothorac Surg 2019 8 06 630 644 31832353 10.21037/acs.2019.10.05 PMC6892713

[6] Dreyfus J Flagiello M Bazire B Isolated tricuspid valve surgery: impact of aetiology and clinical presentation on outcomes Eur Heart J 2020 41 45 4304 4317 32974668 10.1093/eurheartj/ehaa643

[7] Italian Group of Research for Outcome in Cardiac Surgery (GIROC) Di Mauro M Foschi M Dato G MA Surgical treatment of isolated tricuspid valve infective endocarditis: 25-year results from a multicenter registry Int J Cardiol 2019 292 62 67 31130281 10.1016/j.ijcard.2019.05.020

[8] Hall R Shaughnessy M Boll G Drug use and postoperative mortality following valve surgery for infective endocarditis: a systematic review and meta-analysis Clin Infect Dis 2019 69 07 1120 1129 30590480 10.1093/cid/ciy1064 PMC6743840

[9] Lee H A Chou A H Wu V C Nationwide cohort study of tricuspid valve repair versus replacement for infective endocarditis Eur J Cardiothorac Surg 2021 59 04 878 886 33156910 10.1093/ejcts/ezaa390

[10] American Heart Association Committee on Rheumatic Fever, Endocarditis, and Kawasaki Disease of the Council on Cardiovascular Disease in the Young, Council on Clinical Cardiology, Council on Cardiovascular Surgery and Anesthesia, and Stroke Council Baddour L M Wilson W R Bayer A S Infective endocarditis in adults: diagnosis, antimicrobial therapy, and management of complications: a scientific statement for healthcare professionals from the American Heart Association [published correction appears in *Circulation* . 2015 Oct 27;132(17):e215] [published correction appears in *Circulation* . 2016 Aug 23;134(8):e113] [published correction appears in *Circulation* . 2018 Jul 31;138(5):e78–e79] Circulation 2015 132 15 1435 148626373316

[11] Akhtar Y N Walker W A Shakur U Smith G Husnain S S Adigun S F Clinical outcomes of percutaneous debulking of tricuspid valve endocarditis in intravenous drug users Catheter Cardiovasc Interv 2021 97 06 1290 1295 33645916 10.1002/ccd.29584

[12] Iftikhar S F Ahmad F Tricuspid valve endocarditis Treasure Island (FL) StatPearls Publishing 2021 30860694

[13] Moraca R J Moon M R Lawton J S Outcomes of tricuspid valve repair and replacement: a propensity analysis Ann Thorac Surg 2009 87 01 83 88, discussion 88–89 19101275 10.1016/j.athoracsur.2008.10.003

[14] Moss R Munt B Injection drug use and right sided endocarditis Heart 2003 89 05 577 581 12695478 10.1136/heart.89.5.577 PMC1767660

[15] Kang D-H Kim Y-J Kim S-H Early surgery versus conventional treatment for infective endocarditis N Engl J Med 2012 366 26 2466 2473 22738096 10.1056/NEJMoa1112843

[16] Tam D Y Yanagawa B Verma S Early vs late surgery for patients with endocarditis and neurological injury: a systematic review and meta-analysis Can J Cardiol 2018 34 09 1185 1199 30170674 10.1016/j.cjca.2018.05.010

[17] Yanagawa B Elbatarny M Verma S Surgical management of tricuspid valve infective endocarditis: a systematic review and meta-analysis Ann Thorac Surg 2018 106 03 708 714 29750928 10.1016/j.athoracsur.2018.04.012

